# A methodology of theropod print replication utilising the pedal reconstruction of *Australovenator* and a simulated paleo-sediment

**DOI:** 10.7717/peerj.3427

**Published:** 2017-06-06

**Authors:** Matt A. White, Alex G. Cook, Steven J. Rumbold

**Affiliations:** 1 School of Engineering, University of Newcastle, Callaghan, New South Wales, Australia; 2 Palaeontology, Australian Age of Dinosaurs Museum of Natural History, Winton, Queensland, Australia

**Keywords:** *Australovenator*, Megaraptorid, Lark Quarry, Theropod, Trackway

## Abstract

Distinguishing the difference between theropod and ornithopod footprints has proved a difficult task due to their similarities. Herein our aim was to produce a method where a skeleton could be more closely matched to actual fossilised footprints. The reconstructed pes of the Australian Megaraptoran *Australovenator wintonensis* was utilised for this footprint reconstruction. It was 3-D printed in life size, molded and cast to produce a flexible theropod foot for footprint creation. The Dinosaur Stampede National Monument, Lark Quarry, Queensland, Australia was used as our case study to compare fossilised dinosaur footprints with our reconstructed theropod prints. The footprints were created in a sediment that resembled the paleo-sediments of Lark Quarry prior to being traversed by dinosaurs. Measurements of our *Australovenator* prints with two distinctly different print types at Lark Quarry revealed similarities with one distinct trackway which has been the center of recent debate. These footprints consist of 11 consecutive footprints and show distinct similarities in both size and proportions to our *Australovenator* footprints.

## Introduction

Distinguishing theropod tracks from ornithopod tracks has proven a difficult task for ichnologists as both are generally similar. The pes of theropods consist of sharp claws; slender digits; a longer pes opposed to wider pes and a more V-shaped pes outline; whereas ornithopods have hooves (blunt, rounded); wider digits; wider pes proportional to length and a U-shaped pedal outline. These features are easily masked in saturated sediments and unless an actual skeleton is discovered alongside its corresponding prints, speculation can arise regarding the identification of such tracks. An example of this occurring includes the interpretation of one particular bipedal dinosaur trackway at the Dinosaur Stampede National Monument, Lark Quarry, Queensland, Australia (DSNM) (Thulborn & Wade, 1979; Thulborn & Wade, 1984; Thulborn & Wade, 1989; Thulborn, 2013 Romilio & Salisbury, 2011; Romilio & Salisbury, 2014).

Various methods have been developed to analyze the morphology of theropod footprints formed in a variety of sediments with varying fluidity levels, effects of movement including speed and effects on underlying sedimentary layers (e.g., Falkingham, 2014; Falkingham, 2016; Falkingham & Gatesy, 2014; Lallensack, Van-Heteren & Wings, 2016; Milàn & Bromley, 2006; Milàn, 2006). One of the more novel approaches was utilizing live *Dromaius* (emus) to walk through sediments of varying grain sizes and fluidity levels; which demonstrated that morphological variation of the prints were dictated by variations of substrate consistency (Milàn, 2006).

**Figure 1:**
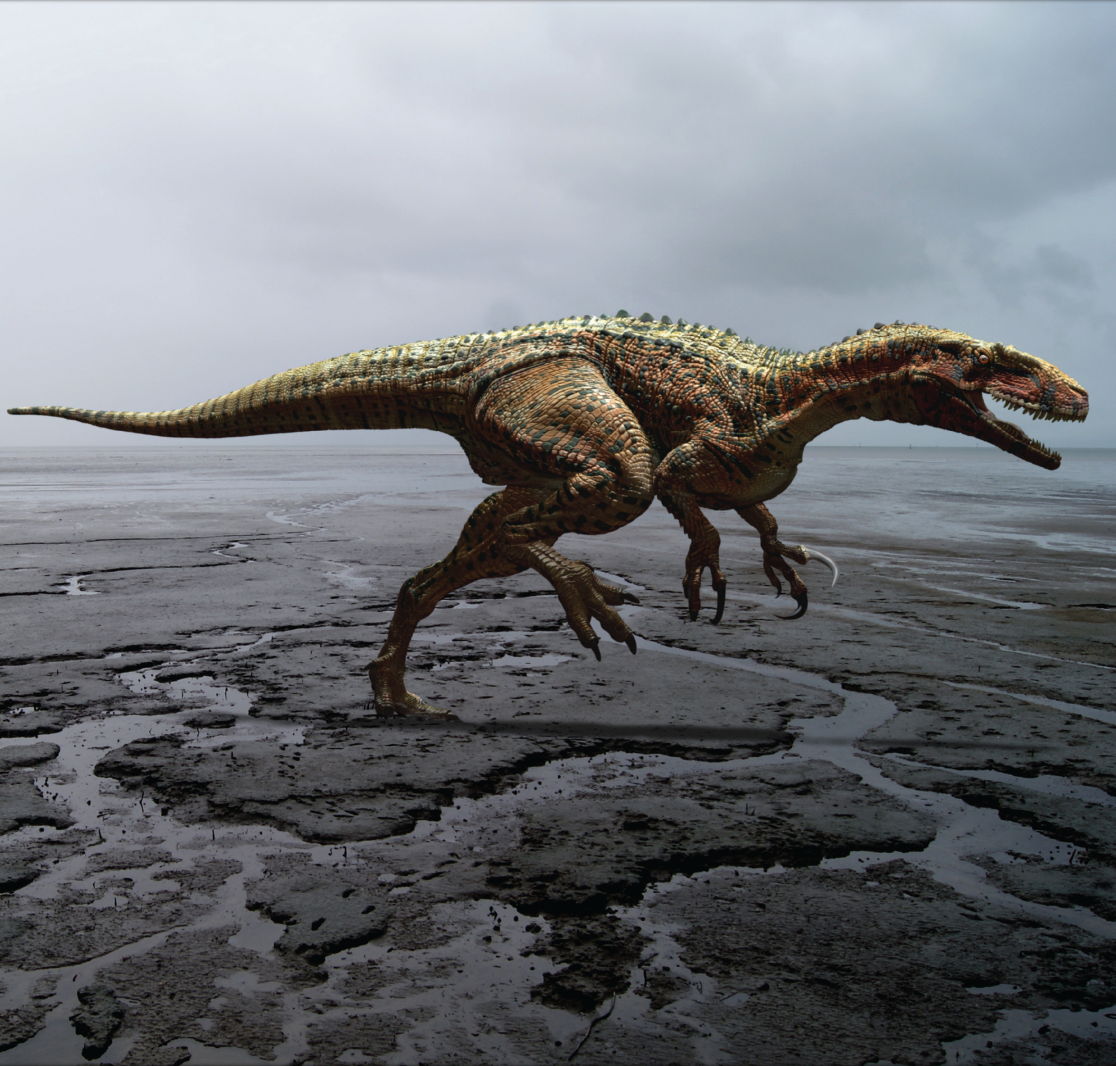
Reconstruction of *Australovenator wintonensis*. Artwork by Travis R. Tischler.

We develop herein a methodology to compare and possibly match a theropod dinosaurs’ pes with a fossilised trackway. This method involved the construction of a 3-D model of a life sized theropod foot (White et al., 2016) which was used to make prints. The only known theropod from Australia, with a complete pes, is *Austrlovenator wintonensis* (Hocknull et al., 2009) (Fig. 1). It was discovered within the same geological formation which has yielded a high number of footprint localities, one of which is the DSNM formally named Lark Quarry. The main objective of this paper is to compare the morphology of the DSNM prints with those made with the 3-D theropod foot model.

## Material and Methods

The *Australovenator* pes restoration and range of motion (ROM) which allowed for the presence of soft tissue, was completed in White et al. (2016). The following methods summarize this work so there is background into how the flexible 3-D *Australovenator* foot prop used to re-create the footprints was created.

### Specimens

**Figure 2:**
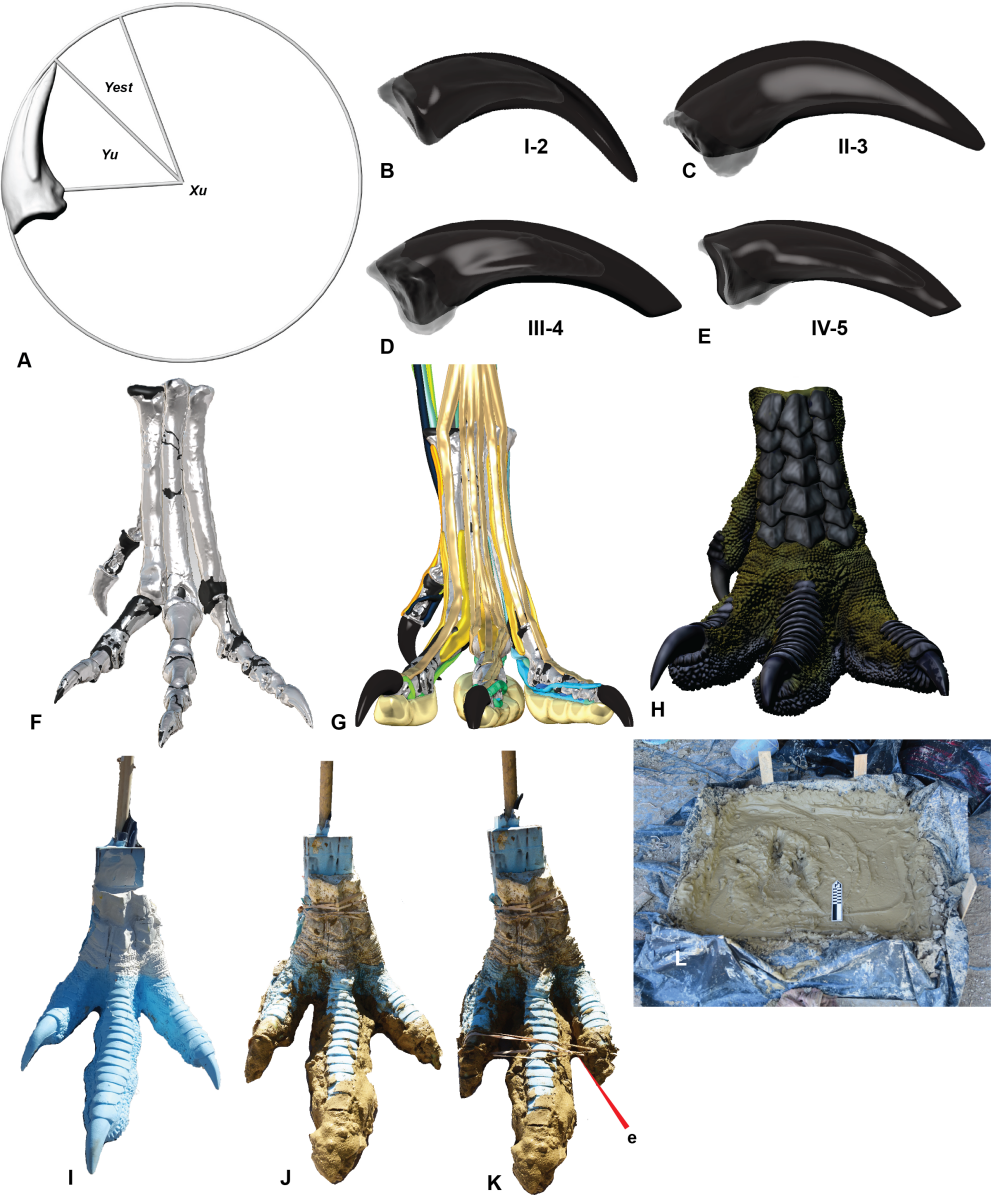
Creation of a theropod print for a comparison with the prints at Lark Quarry using a reconstructed pes of *Australovenator*. (A) Calculating the estimated sheath extent using pedal phalanx IV-5 as an example; (B) Pedal phalanx I-2 with reconstructed sheath; (C) Pedal phalanx II-3 with reconstructed sheath; (D) Pedal phalanx III-4 with reconstructed sheath; (E) Pedal phalanx IV-5 with reconstructed sheath; (F) Reconstructed pes; (G) Biologically reconstructed pes; (H) Skin covered biologically restored pes; (I) Cast of 3-D printed pes using flexible resin; (J) Clay covering the base of the cast foot synthesizing a pes that had already traversed the mud prior to making a subsequent print; (K) The addition of an elastic band to simulate the pes in touch-down articulation; (L) The foot print box used to replicate theropod footprints in various motions and mud at varying saturations.

A complete *Australovenator* pes which pertains to the holotype specimen AODF 604 was used. The specimen included metatarsals I, II, III & IV; pedal phalanges: I-1; I-2; II-1; II-2; II-3; III-1; III-2; III-3; III-4; IV-1; IV-2; IV-3; IV-4; IV-5 (Fig. 2A). Detailed descriptions of each element is provided in White et al. (2013) and White et al. (2016).

The extant phylogenetic bracket of dinosaurs (EPB) introduced by Witmer (1995) is used as this allows for the inference of traits in extinct animals. In this case crocodylians and birds have provided an ‘*in vivo’* tool to determine the effect soft tissue had on the skeletal ROM. We decided to use *Dromaius novaehollandiae* Latham, 1790 (commonly known as an emu) as our ‘*in vivo*’ comparison with the pedal morphology of theropods due to this bird seems to be morphologically closer than crocodiles (White et al., 2016).

The *Dromaius* pes was obtained from an emu farm. It was used to determine its ROM with and without soft tissue. The *Dromaius* specimens used in this analysis include: the distal end of the tarsometatarsus; pedal phalanges: II-1; II-2; II-3; III-1; III-2; III-3; III-4; IV-1; IV-2; IV-3; IV-4; IV-5.

Each pedal element was computed tomography (CT) scanned and the resulting digital imaging and communications in medicine (DICOM) images were converted into 3-D mesh files using Mimics 10.01 software (Materialise HQ, Leuven, Belgium). These files were imported and rearticulated in Zbrush 4R7 3.0 (Pixologic Inc, Los Angeles, CA, USA) (a graphical design package) and Rhinoceros 5.0 (Robert McNeel and Associates, Los Angeles, CA, USA).

### Soft tissue

The *Dromaius* pes was positioned in flexed, extended and weight bearing positions for both computer tomography (CT) scans and magnetic resonance imaging (MRI). The resulting images were converted into separate soft tissue and bone 3-D mesh files which were viewed in Zbrush 4R7 (3.0) and Rhinoceros 5.0 (Robert McNeel and Associates, Los Angeles, CA, USA). The 3-D *Dromaius* meshes were scaled to the same proportional size as the *Australovenator* bone meshes. This provided a guide to digitally articulate the *Australovenator* bones in the same weight-bearing position as the *Dromaius* specimen which enabled an ‘*in silico’* (Hammond, Plavcan & Ward, 2016) restoration of the *Australovenator* pes considering both soft tissue attachment points and proportions (See Fig. 11 in White et al., 2016).

### Claws

The claws of *Australovenator* were well preserved and did not require reconstruction however their corresponding sheaths were not preserved. The extent of where the sheath extended past the tip of the claw (*γ*_est_) originating from the base of the flexor tubercle, can be determined using a formulae that was developed by Glen & Bennett (2007) following their examinations of fossilised claws of dinosaurs, Mesozoic birds and extant birds. The estimated sheath angle was determined by measuring the claw angle of the ungual bone (*γ*_*u*_) and multiplying it by 1.54. The claw angle was determined in Rhinoceros 5.0 from the angle created from the distal limit of the flexor tubercle (*B*_*u*_) and the ungual tip (*T*_*u*_). The resulting formulae is *γ*_est_ = 1.54*γ*_*u*_ (Glen & Bennett, 2007). Cross-sections created through the *Dromaius* sheath and claws revealed that the morphology of the sheath closely matched the underlying claw. Therefore we restored the *Australovenator* sheaths to match the morphology to the underlying bone (Figs. 2A–2E; Table 1).

**Table 1:** Estimated sheath extents utilisingthe formulae *Y*_est_ = 1.54*Y*_*u*_.

Claw	*γ*_*u*_	*γ*_*est*_
MTI-2	64	99
MTII-3	71	109
MTIII-4	53	82
MTIV-5	48	73

**Notes.**

Abbreviations Y_est_ Estimated angle of the sheath Y_u_ claw angle of the ungual bone

### Range of motion

The ROM of the *Australovenator* pes was determined from a comparative ROM analysis of the extant cursorial bird *Dromaius novaehollandiae* Latham, 1790, with and without soft tissue. The variance of ROM with and without soft tissue was applied to the ROM of the *Australovenator* pes which provided an estimation for the soft tissue ROM (White et al., 2016).

The soft tissue ROM of *Dromaius* was achieved by bending the digits to their maximum flexion and extension limits and securing them in place for CT scanning. The pes was also poised with the metatarsus in a vertical position simulating a weight bearing phase on a flat substrate to represent a neutral (weight-bearing) position (0°). This reduced the extension values and increased the flexion values however the total ROM capability is the addition of extension and flexion values.

**Figure 3:**
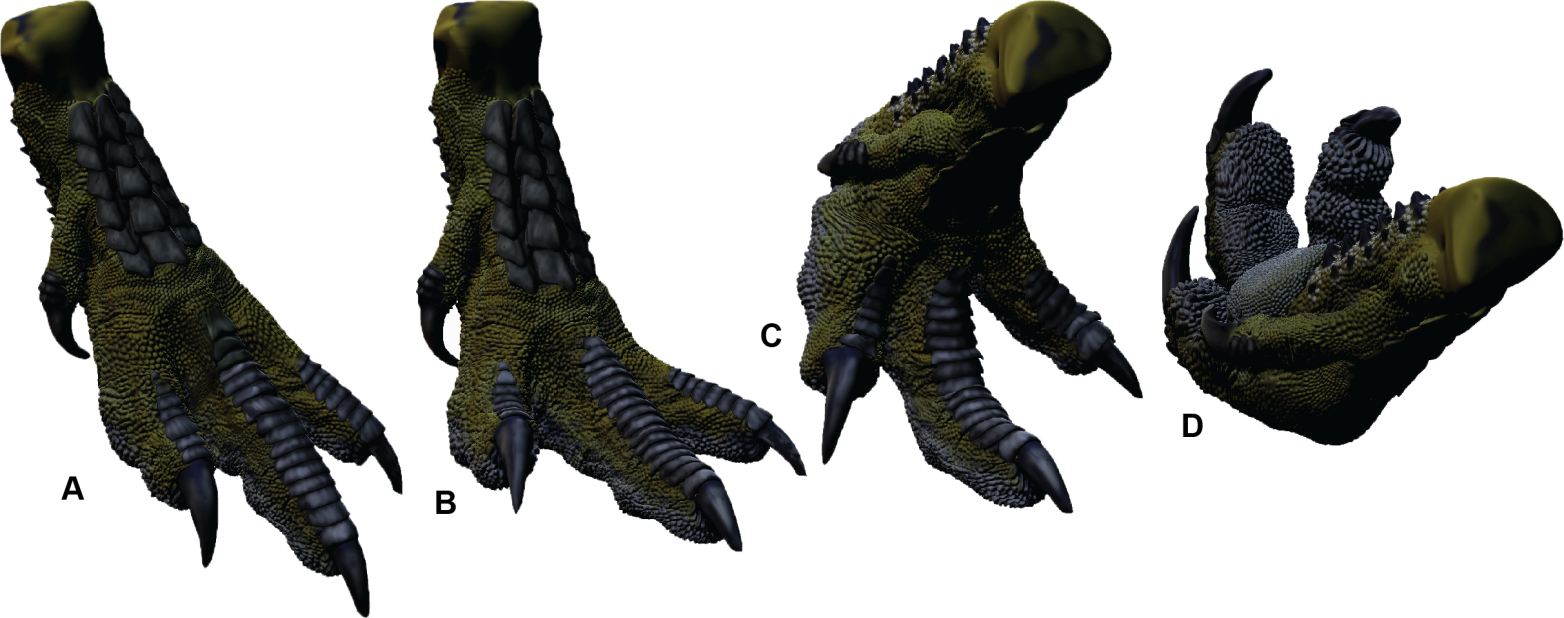
Range of motion of the * Australovenator* pes. (A) Touch-down phase; (B) Weight-bearing phase; (C) Kick-off phase; (D) Suspended phase following kick-off.

To determine the *Dromaius* bone ROM, the soft tissue was removed and the bones were CT scanned individually. The separate 3-D meshes were imported into Zbrush 4R7 where they were articulated to their ROM limitations using the *Dromaius* bones as manual references (see Fig. 5 in White et al., 2016). The variation between the bone and soft-tissue ROM was converted to a percentage which was used to later infer the soft tissue ROM of the *Australovenator* pes. The extension and flexion ROM limits were multiplied by the ROM variation percentage of the *Dromaius* pes in order to provide an estimation of the soft-tissue ROM for *Australovenator* (Fig. 3).

### 3-D Flexible *Australovenator* Foot Manufacturing

*Australovenator* soft-tissue pes was reconstructed *in silico* in the weight-bearing position (Fig. 3B). The soft-tissue proportions were established by creating 3-D meshes of the *Dromaius* pes with and without soft tissue. These meshes were rescaled individually or together in both Rhinoceros 5.0 and Zbrush 4R7 so the soft-tissue mesh was scaled along the *Dromaius* bones. The *Dromaius* bone meshes were scaled to match the size of the *Australovenator* bone meshes. The *Dromaius* soft tissue mesh provided an *in silico* 3-D outline guide for reconstructing the *Australovenator* soft tissue. The detailed description of each muscle group was provided in White et al. (2016). The skin texture was restored based on the preserved skin of *Concavenator corcovatus* (Ortega, Escaso & Sanz, 2010) from the Lower Cretaceous of Cuenca, Spain (Cuesta et al., 2015) (Fig. 2H). They share a common ancestor along the Carcharodontosaurian lineage as per a recent phylogeny provided in Cuesta et al. (2015).

In order to make a physically flexible *Australovenator* foot, the 3-D mesh was sectioned into smaller parts to fit into a 3-D printer using Rhinoceros 5.0. These ridged solids were assembled to form a life sized *Australovenator* pes. This solid pes was then moulded using Flexithane 40 and then cast using SRT-30 Silicone. The silicone cured into a flexible rubber to resemble the estimated ROM achievable with soft tissue reported in White et al. (2016) (Fig. 2I). The rubber also allowed slight flattening of the digital pads as what occurs in an actual foot (see Fig. 5 in Gatesy, 2001).

The claws were initially made of the same flexible rubber as the rest of the cast; however this was overcome by coating them with araldite glue, which formed a ridged shell resembling keratin sheaths. We used elastic bands to bring digits II and IV closer to digit three (Fig. 2K). This structure enabled us to simulate a suspended articulation prior to making contact with the substrate. During touch-down the elastic bands were severed and the digits returned to their weight-bearing phase posture. The pes was then rolled forward through the substrate simulating the kick-off phase (Fig. 3C).

### Paleo-environment and sediment replication for footprint recreation

The stratigraphic sequence of Lark Quarry was initially interpreted as lacustrine and fluviatile in origin (Thulborn & Wade, 1984). Following the periodic influxes of water, fine cross-bedded sands were deposited. These were covered by clay when the water turbulence subsided. The footprints were impressed in a seam of colour banded claystone which was underlain by cross-bedded sandstones (Thulborn & Wade, 1984; Thulborn, 2017).

The sediment we used to make the *Australovenator* prints, was mixed in order to simulate the palaeo-environment of DSNM prior to being traversed by dinosaurs. The claystone that houses the footprints was replicated by utilising clay from the dinosaur bone-beds from the surrounding areas. The clay was first dried and then saturated with water. Underlying the claystone was fine-grained cross-bedded sandstones (Thulborn & Wade, 1984; Thulborn, 2017) which were replicated with fine-grained sand. The sediment was layered in a 1 × 1 m sediment box which was lined with plastic. The fine-grained sand formed the base, which was overlain with the highly saturated clay (Fig. 2L).

### Photogrammetry

The *in situ* Lark Quarry **‘**bipedal’ prints and the replicated *Australovenator* prints were digitised in 3-D using Agisoft Photo Scan. A Nikon 810D was used to take the photographs with both a 10–24 mm and 105 mm lens. The 3-D meshes were viewed in both Agisoft and Rhinoceros 5.0. The Lark Quarry tracks were photographed shortly after they were cleaned in early 2016.

### Measurements

The measurements that are included here were taken using the software package Rhinoceros 5.0. Scale photographs were taken and imported as a background image. This was used to scale the 3-D mesh of the footprint created in Agisoft Photo Scan so accurate measurements could be taken in Rhinoceros 5.0.

## Results

### Recreating the prints of *Australovenator*

The *Australovenator* footprints were made by the flexible foot simulating the phases of touch-down, weight bearing and kick-off (Fig. 3). Creating the footprints took place during several days where the clay was re-saturated at the beginning of each day and by days end had begun to dry out. Subsequently the prints were formed in varying levels of saturation throughout the day which diversified the data set. The highly saturated clay collapsed inward either shortening or narrowing the digits, whereas lesser saturated clay created a distinct print resembling the flexible foot. Following the creation and photography of each footprint the clay was churned over erasing the print, smoothed over to resemble a flat newly deposited clay ready for the following imprint.

**Figure 4:**
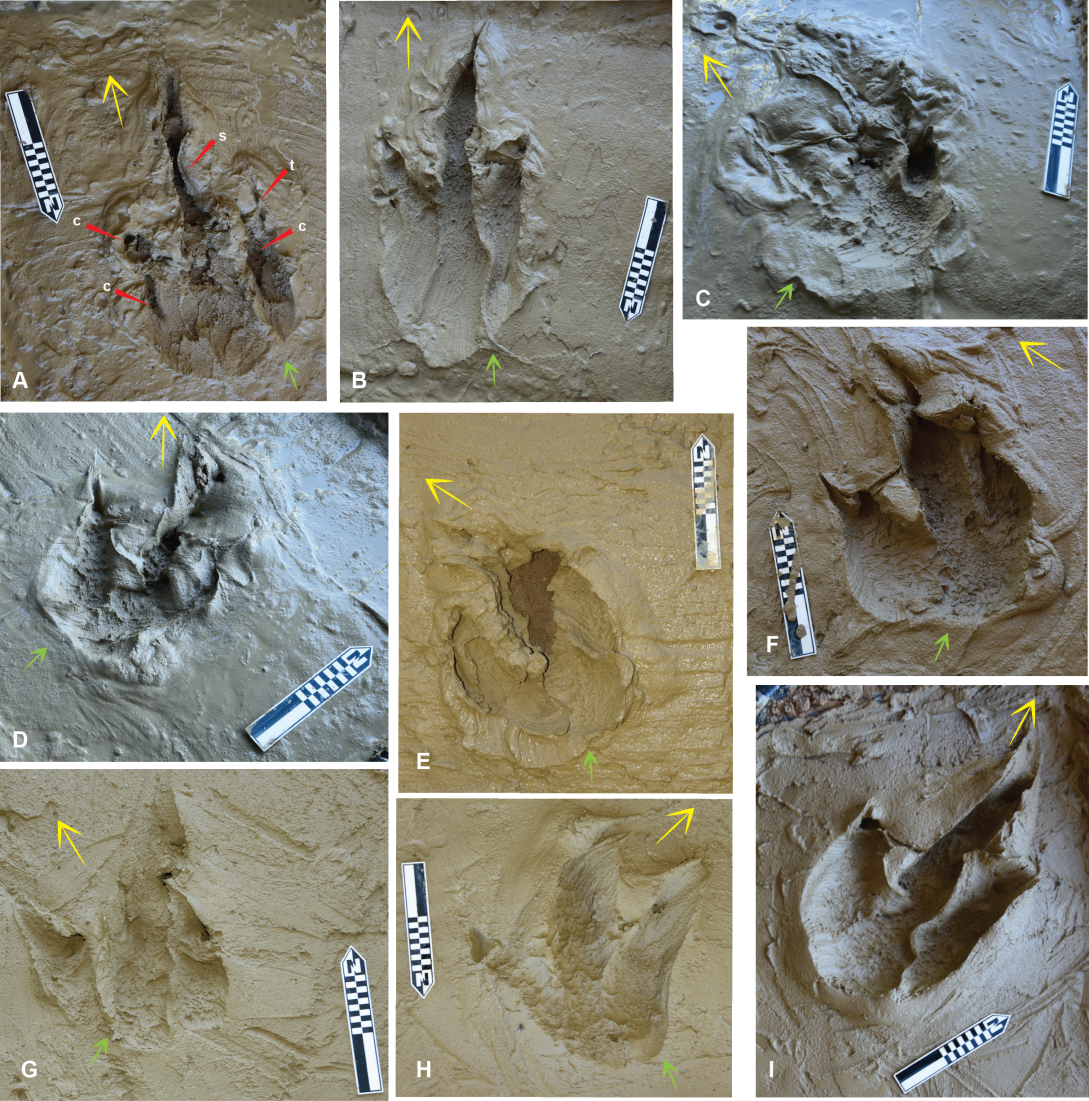
The *Australovenator* prints that were measured and compared with prints from the DSNM Lark Quarry. Symbols: small arrow entry direction; large arrow exit direction. (A) Pes forward motion; (B) Pes forward motion with heel slide; (C) Pes entered substrate at a slight angle favoring the medial side with digit IV entering substrate first, followed by full weight bearing phase, slight rotation and exiting substrate veering left; (D) Pes entered substrate at slight angle favoring the lateral side with digit IV entering substrate first, followed by full weight bearing phase, slight rotation and exiting substrate veering left, with sediment bulging up on the medial side of the heel; (E) Pes entered substrate in forward motion with digit V contacting substrate first followed by full weight bearing phase and then was rotated *in situ* simulating a direction change veering left; (F) Pes entered substrate at a slight angle favoring the lateral side with digit IV entering substrate first, followed by full weight bearing phase, slight rotation and exiting substrate veering left, with sediment bulging up on the medial side of the heel; (G) Pes entered substrate at a slight angle favoring the lateral side with digit IV entering substrate first, the heel did not contact the sediment and the pes exited the substrate veering left; (H) Pes entered substrate at a slight angle favoring the medial side with digit II making first contact with the substrate, the heel did not contact the sediment as the pes exited the substrate veering right, which resulted in a shallow digit IV impression; (I) Pes entered substrate in a forward motion followed by full weight bearing phase and exited the substrate in the same direction. Abbreviations: claw (c); slumping (s); tunnel (t).

**Figure 5:**
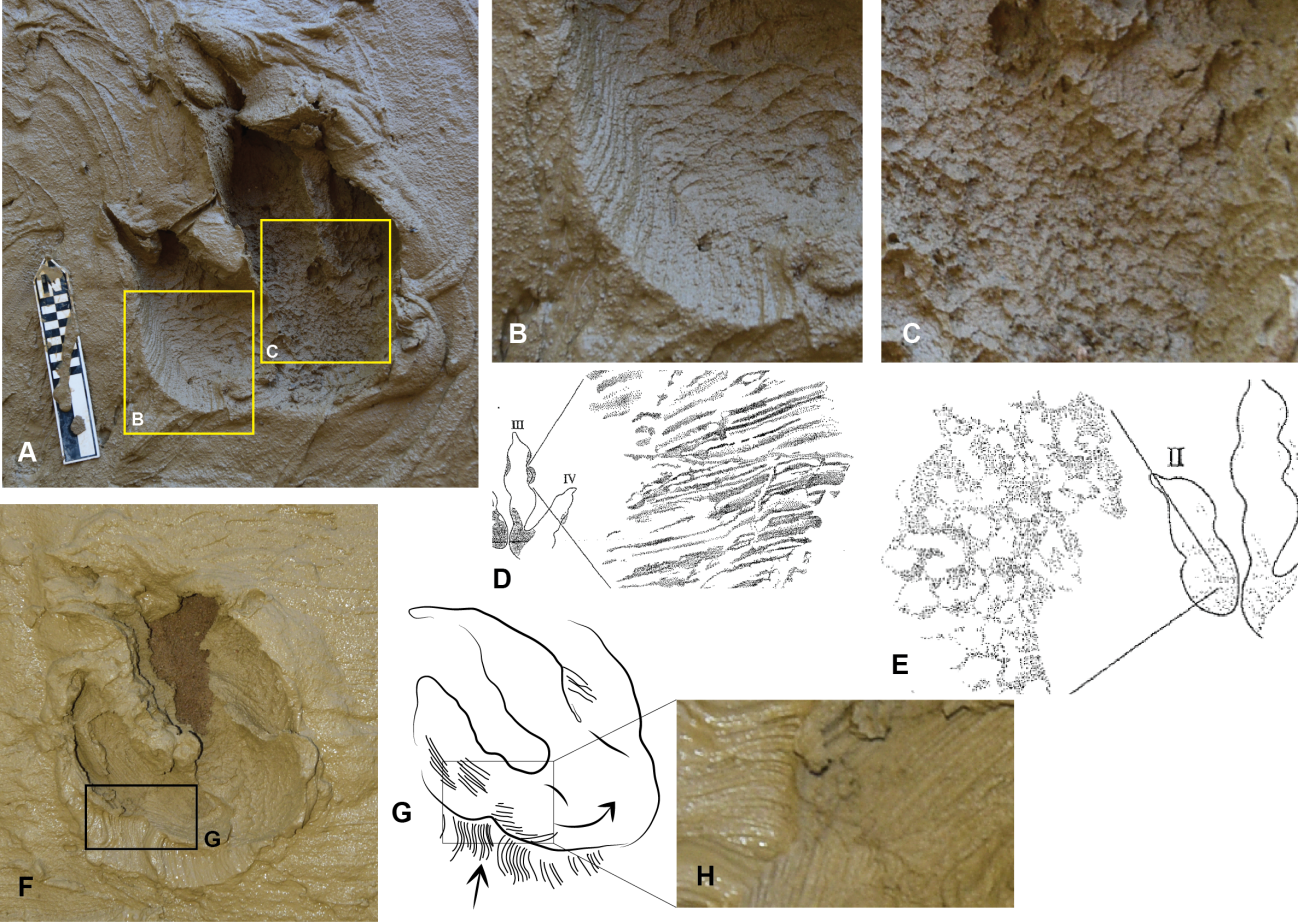
Trace features created by the *Australovenator* flexible foot prop. (A) Left *Australovenator* print displaying entry striation grooves (B) and skin impressions created from the papillae from the base of the foot (C); (B) close-up of the parallel grooves created from the *Australovenator* foot entering the sediment; (C) Close-up of the digital pad impression formed by the rounded papillae; (D) Fossilised Triassic theropod prints with parallel striation grooves (adopted from Fig. 1D in Gatesy, 2001); (E) Fossilised Triassic theropod prints with pad impressions displaying hexagonally arranged dimples (adopted from Fig. 1A in Gatesy, 2001); (F) *Australovenator* print displaying entry striation grooves and curved striations created from the papillae as the pes twisting in the sediment to initial a change in direction; (G) Outline of striations draw from (F); (H) Close up of striations in (F).

The *Australovenator* flexible foot was forced into the clay at various angles to simulate movement in: a forward direction, a turn to the left and a turn to the right. The left turn resulted in sediment pushed up between digits III and IV (Figs. 4F and 4G) whereas a turn to the right pushed up sediment between digits II and III (Fig. 4H). When the flexible foot prop was forced into the sediment at an angle to simulate a left or right turn it occasionally created parallel grooves in the sediment formed by the replicated skin papillae (Figs. 5A, 5F). When the foot prop was turned in the sediment the digits and heals created curved parallel striations. The digital pad and heal impressions were a mixture of semi-rounded to hexagon convex impressions. When the foot prop was rolled through the sediment it resulted in some sediment suction which created various sediment suction peaks in the prints. The digital pad impressions showed remarkable similarities to theropod skin impressions that were described in Gatesy (2001) (Figs. 5D, 5E). These included densely packed convex hexagon like depressions of the prints base and regions of parallel grooves, which Gatesy (2001) identified as entry striations created by the skins papillae and as the theropods foot entered the sediment.

**Figure 6:**
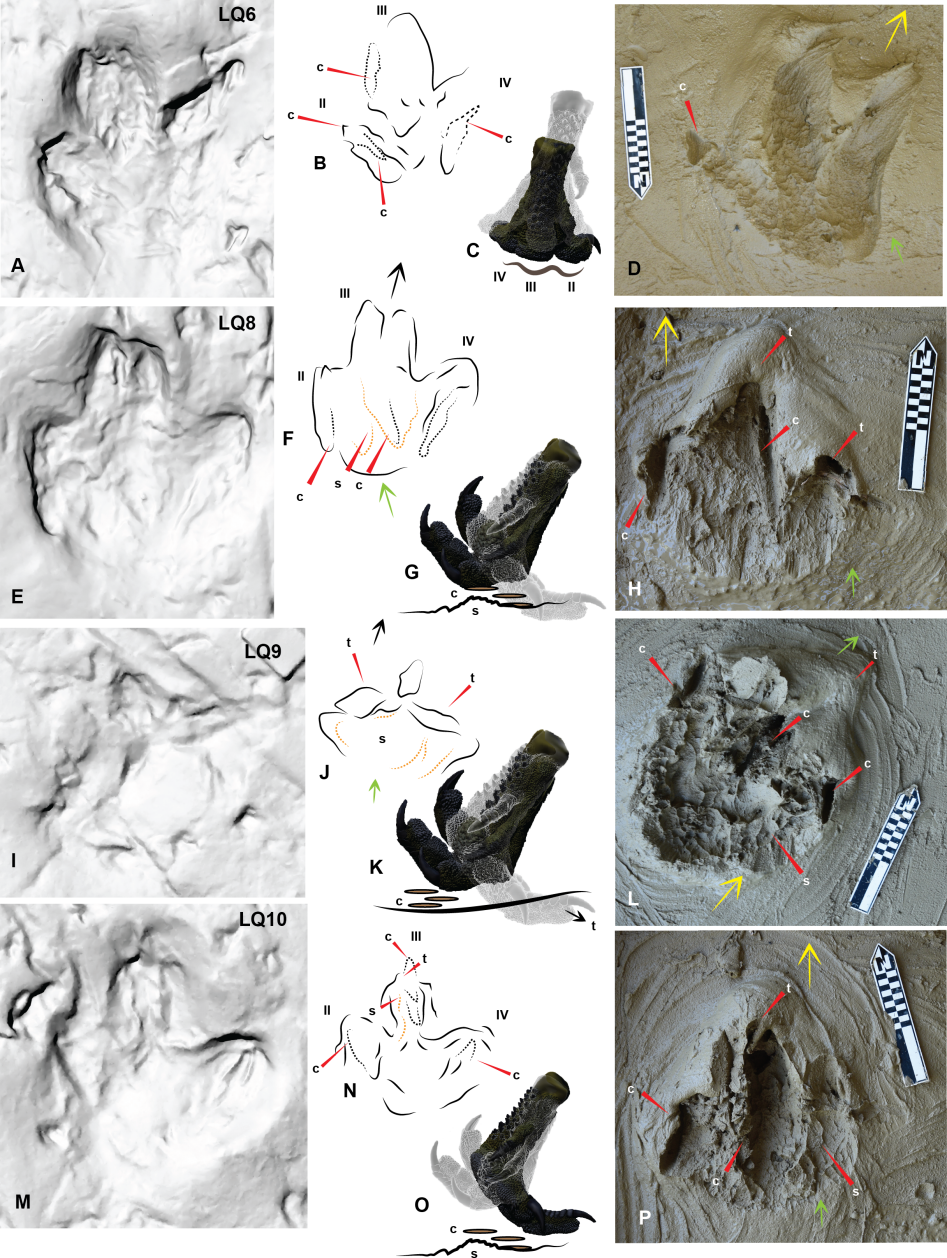
Comparing Trackmaker A prints of Lark Quarry with the *Australovenator* prints to help identify various morphological features of the Lark Quarry prints. (A) Trackmaker A LQ6 (right) a digitigrade print slightly favoring the lateral side; (B) Trace of morphological features and print outline of LQ6; (C) Graphic depicting the movement of the *Australovenator* foot (left foot) used to create (D); (D) *Australovenator* print simulated by favoring the medial side which resulted in digits II and III to be slightly deeper than digit IV, similar to LQ6; (E) Trackmaker A LQ8 (right) a digitigrade print with a distinct claw trace of digit II and a slight claw trace of digit IV; (F) Trace of morphological features and print outline of LQ8; (G) Graphic depicting the movement of the *Australovenator* foot used to create (H); (H) *Australovenator* digitigrade print which resulted in some minor tunneling of the digits before they were manually flicked backward creating claw traces; (I) Trackmaker A LQ9 (left) a shallow digitigrade print; (J) Trace of morphological features and print outline of LQ9; (K) Graphic depicting the movement of the *Australovenator* foot used to create (L); (L) *Australovenator* digitigrade print with digit tunneling, which if they collapsed, would make the print similar to LQ9; The sediment was slightly drier than the sediment used to create (D) which created more sediment suction, resulting in pronounced hexagonal like papillae traces; (M) Trackmaker A LQ10 (right) digitigrade print with slight digit tunneling and claw traces of each digit; (N) Trace of morphological features and print outline of LQ10; (O) Graphic depicting the movement of the *Australovenator* foot used to create (P); (Q) *Australovenator* digitigrade print with minor digit tunneling and kick-off claw traces similar to LQ10. Abbreviations: claw trace (c), suction of substrate to the foot causing suction trace (s), tunnel features of the digits (t). Arrows depict direction of movement.

The claws and some of the digits occasionally formed tunnels in the sediment when the pes was forced forward. Varying the direction the pes was forced into and rolled out of the sediment was to simulate different movements and direction change of a theropod. Exaggerated heel slides resulted in slightly longer prints (Fig. 4B), prints making a left turn resulted in a shallower digit II impression than digit IV (Figs. 4D, 4F and 4G) whereas during a right turn digit II was deeper (Fig. 4H). The simulated digitigrade footprints created tunnel like features in the substrate with the proximal portion of the footprint tapering towards the digit impressions (Figs. 6H, 6L and 6P).

### *Australovenator* print measurements and morphology

**Figure 7:**
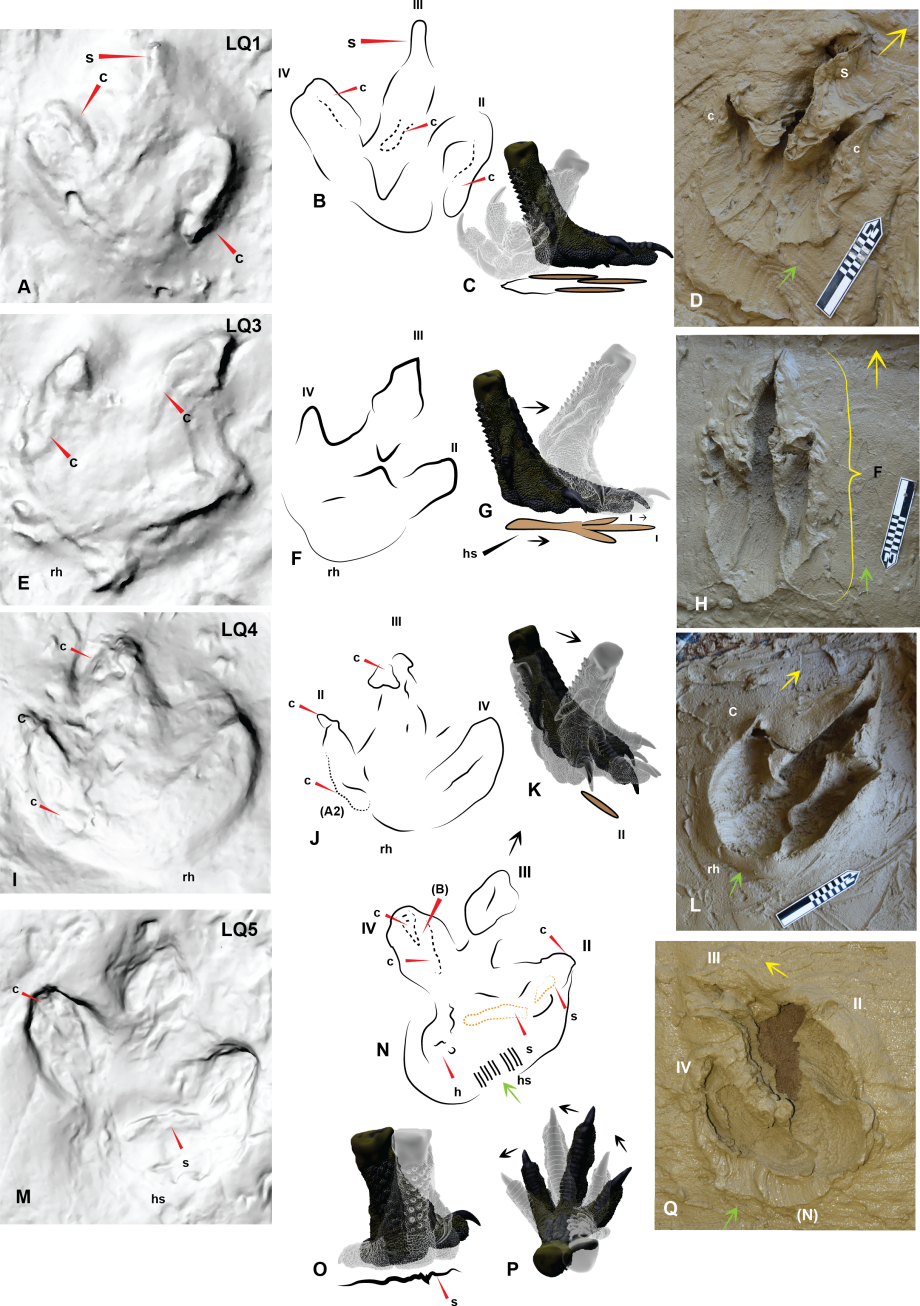
Comparing Trackmaker A prints of Lark Quarry with the *Australovenator* prints to help identify various morphological features of the Lark Quarry prints. (A) Trackmaker A LQ1 (left foot), with a distinct claw trace of digit II; (B) Trace of morphological features and print outline of LQ1; (C) Graphic depicting the movement of the *Australovenator* foot used to create (D); (D) *Australovenator* print with a full heel contact similar to LQ1; (E) Trackmaker A LQ3 (left foot) is the longest print of this trackway; (F) Trace of morphological features and print outline of LQ3; (G) Graphic depicting the movement of the *Australovenator* foot used to create (H); (H) *Australovenator* print with a slight heel slide creating a slightly longer print similar to LQ3; (I) Trackmaker A LQ4 (right foot) has been restored *in situ* around the heel region and digit IV giving the heel a rounded appearance; (J) Trace of morphological features and print outline of LQ4; (K) Graphic depicting the movement of the *Australovenator* foot used to create (L); (L) *Australovenator* print with full heel contact, favoring the lateral side creating a wider fourth digit impression, which formed a rounded heel impression (generally regarded as characteristic of ornithopod prints) which is similar to LQ4; (M) Trackmaker A LQ5 (left foot) favored the medial side when entered into the substrate with an exaggerated heel slide. Some claw traces following kick-off are also visible; (N) Trace of morphological features and print outline of LQ5; (O) Graphic depicting the movement of the *Australovenator* foot used to create (P); (P) *Australovenator* print favoring the lateral side with full heel contact and slight rotation to simulate direction change. Abbreviations: claw trace (c), suction of substrate to the foot causing suction trace (s), tunnel features of the digits (t). Arrows depict direction of movement.

**Figure 8:**
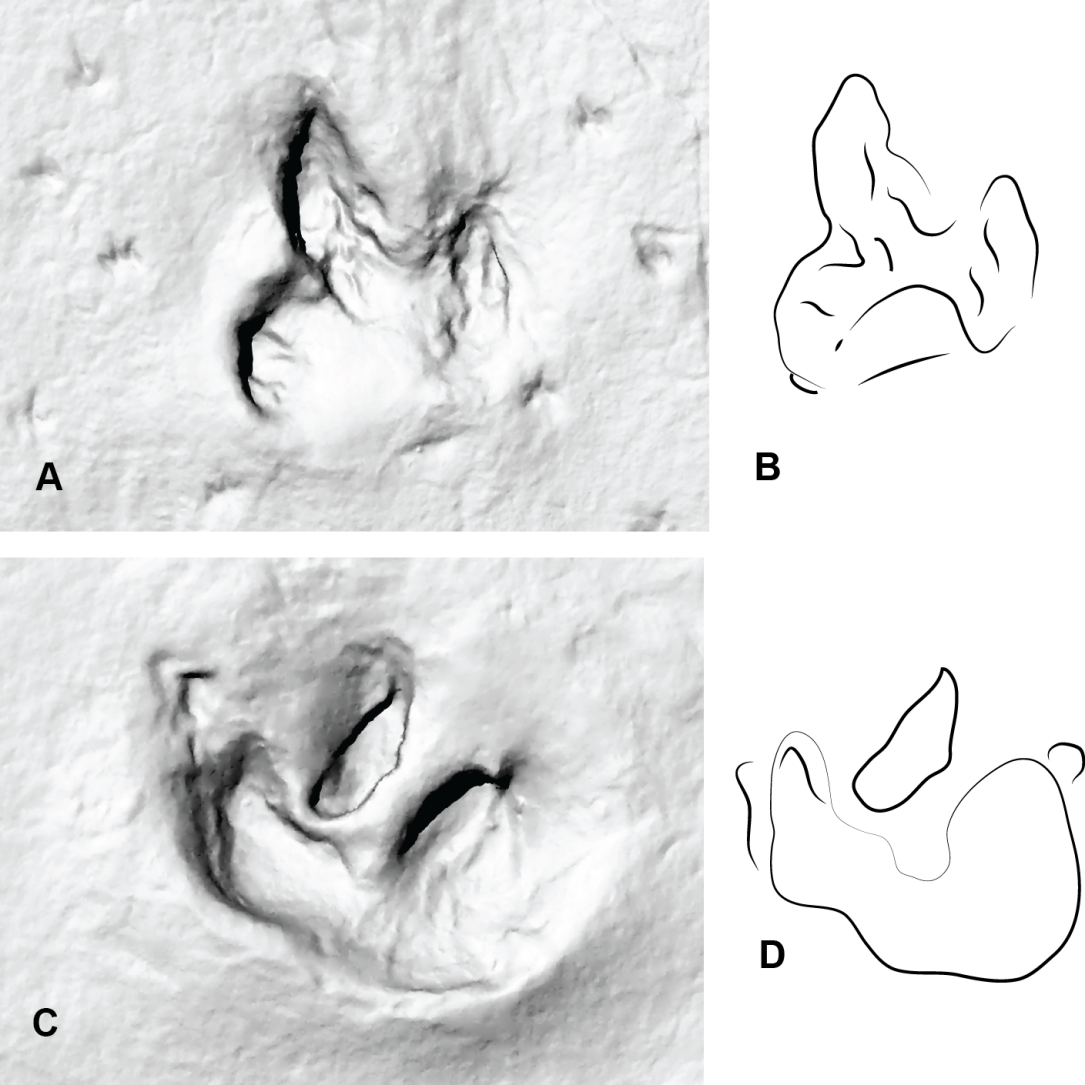
Two consecutive prints from Trackmaker B (ornithopod) from DSNM Lark Quarry. (A) Print 3 (left); (B) Trace of print 3; (C) Print 4 (right); (D) Trace of print 4.

**Figure 9:**
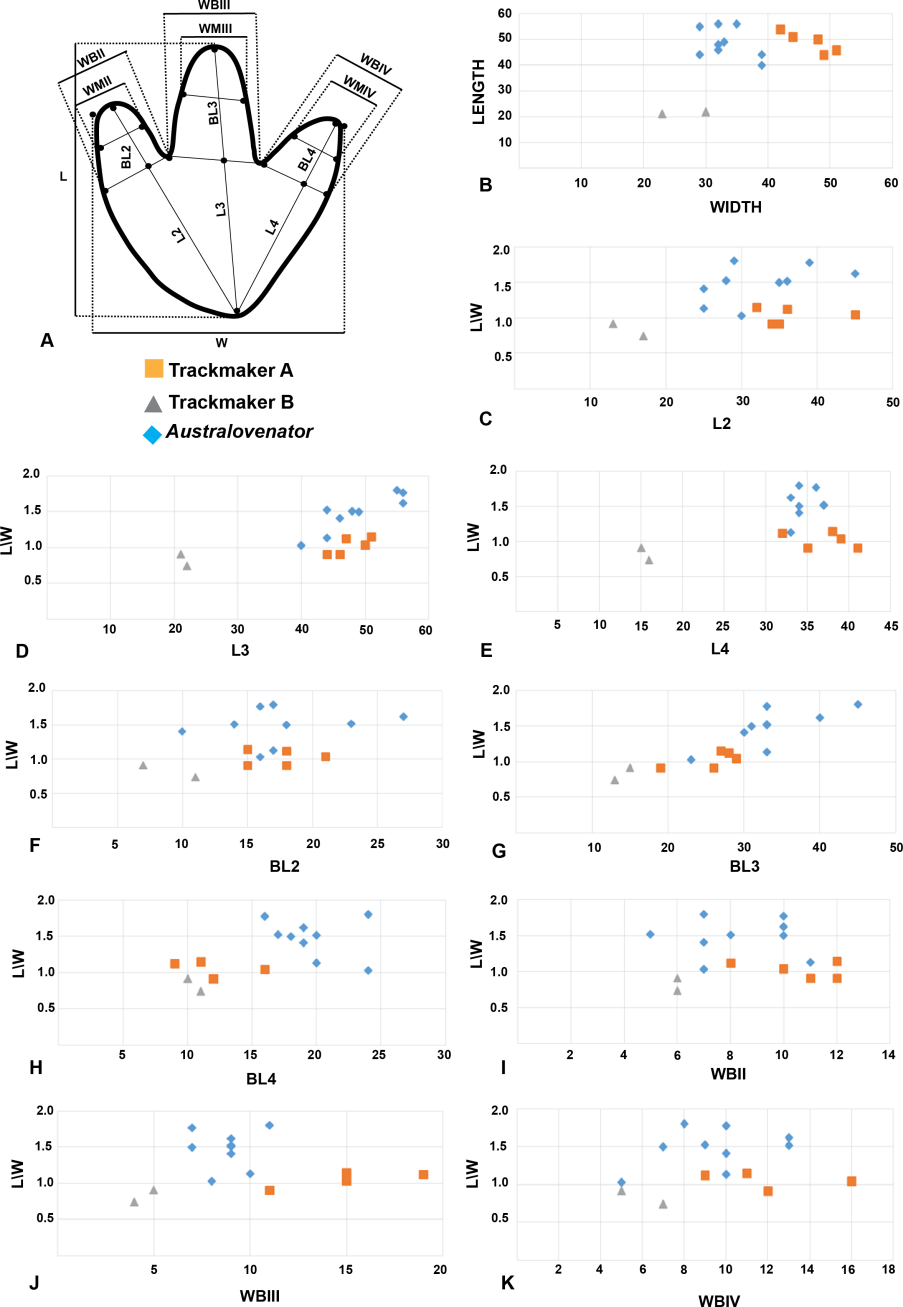
Bivariate plots comparing the print proportions of Trackmaker A, Trackmaker B and *Australovenator*. (A) Measurement diagram; (B) Length against Width; (C) (L/W) against (L2); (D) (L/W) against (L3); (E) (L/W) against (L4); (F) (L/W) against (BL2); (G) (L/W) against (BL3); (H) (L/W) against (BL4); (I) (L/W) against (WBII); (J) (L/W) against (WBIII); (K) (L/W) against (WBIV).

Measurements of nine *Australovenator* prints (Fig. 4) were averaged and compared with a sample of tracks from DSNM. These included the first five prints from a trackway of 11 prints which were identified as a ‘carnosaur’ in Thulborn & Wade (1984) and an ornithopod in Romilio & Salisbury (2011) here referred to as Trackmaker A (Fig. 7); and two prints from a trackway of 8 prints of a distinctly different bipedal dinosaur (ornithopod in Thulborn & Wade, 1984) here referred to as Trackmaker B (Fig. 8; Table 2). A bivariate analysis was employed to help distinguish the variance between these trackmakers. The parameters used in this analysis include; length (L); width (W); total digit lengths (LII-IV); basal digit lengths (BL2-4); basal digit widths (WBII-IV); middle digit width (WMII-IV). These measurement parameters were adopted from the work of Moratalla, Sanz & Jimenez (1988) who initially employed the method to distinguish between theropod and ornithopod prints. Each parameter was plotted against (L/W) to compare individual parameters with the overall proportional size of the print in question (Fig. 9).

The results of the plots showed that the parameters of Trackmaker A and *Australovenator* congregated whereas the parameters of Trackmaker B were distinct outliers (Fig. 9; Tables 2–3). The only variation from this was the BLIV which showed a clustering of Trackmaker A & B values. The digit width parameters (WBII-IV; WMII-IV) in this analysis did not reveal any distinct separation between the three print types. The clustering of the Trackmaker A with the *Australovenator* values indicates that its parameters were closer to the prints of a theropod where as Trackmaker B was distinctly different.

**Table 2:** Trackmaker B prints 3–4 measurements.

Print no.	L	W	L/W	D2	D3	D4	BL2	BL3	BL4	WBII	WBIII	WBIV	WMII	WMIII	WMIV
Print 3	21	23	0.91	13	21	15	7	15	10	6	5	**5**	4	7	4
Print 4	22	30	0.74	17	22	16	11	13	11	6	4	**7**	10	5	6
Average	21.5	26.5	0.8	15.0	21.5	15.5	9.0	14.0	10.5	6.0	4.5	6.0	7.0	6.0	5.0

**Notes.**

Abbreviations L Length W Width D Digit BL2 base digit 2 length BL3 base digit 3 length BL4 base digit 4 length WBII base of digit 2 width WBIII base of digit 3 width WBIV base of digit 4 width WMII digit 2 mid width WMIII digit 3 mid width WMIV digit IV mid width

**Table 3:** *Australovenator* (theropod) print measurements of Fig. 4.

Figure 4	L	W	L/W	D2	D3	D4	BL2	BL3	BL4	WB2	WB3	WB4	WM2	WM3	WM4
A	55	29	1.80	29	55	34	17	45	24	7	11	8	5	5	6
B	56	32	1.77	39	56	36	16	33	16	10	7	10	5	7	8
C	46	32	1.41	25	46	34	10	30	19	7	9	10	6	4	5
D	44	29	1.52	28	44	37	23	33	17	5	9	9	4	6	6
E	49	33	1.50	35	49	34	18	31	18	10	7	7	7	7	8
F	48	32	1.51	36	48	37	14	33	20	8	9	13	6	9	7
G	44	39	1.13	25	44	33	17	33	20	11	10	10	9	8	8
H	40	39	1.03	30	40	39	16	23	24	7	8	5	6	7	4
I	56	35	1.62	45	56	33	27	40	19	10	9	13	10	8	12
AVERAGE	49	34	1.45	32	49	35	17	34	20	8	9	10	6	7	7

**Notes.**

Abbreviations L Length W Width D Digit BL2 base digit 2 length BL3 base digit 3 length BL4 base digit 4 length WBII base of digit 2 width WBIII base of digit 3 width WBIV base of digit 4 width WMII digit 2 mid width WMIII digit 3 mid width WMIV digit IV mid width

The following aspects were also considered with both print types which included: length verses width of the prints with longer prints generally attributed to theropod and wider prints ornithopod (Lockley, 2009) (Fig. 9B); the shape of the claws (sharp or rounded); overall configuration of the foot print (V-shaped in theropods, U-shaped in ornithopods). Interestingly it was demonstrated by Milàn (2006) that the footprint morphology varies greatly depending on the sediment composition and fluid saturation which was subsequently demonstrated with our *Australovenator* prints. Some prints exhibited U-shaped configurations characteristic of ornithopods (Figs. 4B, 4C, 4E, 4G, 4I). Additionally sharp claw outlines were not always visible (Figs. 4I, 6H, 6L and 7Q).

## Discussion

The DSNM Trackmaker (A) shares a similar size, morphology and configuration to the *Australovenator* prints. They are generally longer than wider, some possess sharp claw marks and the actual digital lengths resemble the average measurements of the *Australovenator* prints (Tables 3 and 4). The DSNM Trackmaker (B) does not share any similarities with either the *Australovenator* prints or Trackmaker (A). Its print is distinctly wider than longer and the overall configuration is U-shaped (Fig. 8).

The *Australovenator* digitigrade footprints were created with minimal to no heal contact with the substrate. Various features of these footprints resembled some of the footprints from Trackmaker (A) where it transitioned from a full heal impression in print 5 to digitigrade prints from 6–11 (Figs. 6E–6M). These included tunnel features as the digits were forced down into the substrate; claw flick marks as the pes exited the substrate; and a tapering heal toward the digit impressions. To create the claw flick marks the pes was manually flicked backward through the sediment following the initial digitigrade print simulating the kick-off phase. The resulting traces left by the claws are similar to features that are present in some of the DSNM Lark Quarry prints (Figs. 6E–6M).

The DSNM was a perfect case study to test this methodology as there has been some speculation regarding the identity of some of the trackmakers (i.e., Romilio & Salisbury, 2011; Romilio, Tucker & Salisbury, 2013; Romilio & Salisbury, 2014. More specifically the 11 consecutive prints originally identified as a ‘carnosaur’ in Thulborn & Wade (1979) and Thulborn & Wade (1984) was speculated to actually be an ornithopod in Romilio & Salisbury (2011) and Romilio & Salisbury (2014). Comparing the DSNM prints with our *Australovenator* prints revealed distinct similarities in proportions and morphology (Tables 3 and 4). Variations of the *Australovenator* print morphology reveals the importance of not solely relying on information from individual prints (i.e., presence of claw marks; print configuration (U or V shaped) and the length and width of the prints). Interestingly the average proportions of the *Australovenator* prints closely matches with the average size and digit proportions of bipedal Trackmaker A. These average measurements suggests that the Trackmaker A resembles a theropod rather than an ornithopod. Due to this similarity, various *Australovenator* prop prints were used to identify various morphological features of Trackmaker A (Figs. 6 and 7). The ROM that was used to create the *Australovenator* prints is depicted in (Figs. 6C, 6G, 6K, 6O, 7C, 7G, 7K, 7O, 7P).

**Table 4:** Trackmaker A print measurements of prints 1–5.

Print no.	L	W	L/W	D2	D3	D4	BL2	BL3	BL4	WB2	WB3	WB4	WM2	WM3	WM4
1	51	44	1.15	32	51	38	15	27	16	12	15	**11**	10	9	11
2	50	48	1.04	45	50	39	21	29	16	10	15	16	9	11	17
3	**54**	42	1.12	36	47	32	18	28	11	8	19	**9**	7	10	7
4^a^	46	51	0.91	35	46	41	15	26		11			8		
5	44	49	0.91	34	44	35	18	19	19	12	11	12	7	11	14
Average	49	46	1.07	37	48	36	18	26	15	11	15	12	8	10	12

**Notes.**

Abbreviations L Length W Width D Digit BL2 base digit 2 length BL3 base digit 3 length BL4 base digit 4 length WBII base of digit 2 width WBIII base of digit 3 width WBIV base of digit 4 width WMII digit 2 mid width WMIII digit 3 mid width WMIV digit IV mid width

a Prints distorted by other Lark Quarry trackmakers.

The identity of the trackway maker along the southern portion of DSNM (Trackmaker B) has not been debated from its original identification as an exceptionally large ornithopod (Thulborn & Wade, 1984). Trackmaker B’s average measurements depicted in the bivariate plots distinctly segregate them from Trackmaker A (Fig. 9, Tables 2 and 4). Additional supporting feature are the prints are distinctly wider than they are long and have rounded U-shaped or flattish heal impressions. However, as previously noted, these features alone cannot be solely used to distinguish ornithopod from theropod. The variation in shape and measurement proportions strongly suggest that the authors of Trackways A and B were two different bipedal dinosaurs.

## Conclusion

Our case study of utilising the *Australovenator* pes to re-create theropod prints, enabled an effective comparison with various prints at DSNM Lark Quarry. Our re-created *Australovenator* prints displayed similar proportions, shape and overall size to a recently debated trackway that we referred to as Trackmaker A, consisting of 11 consecutive prints. Based on our analysis the Trackmaker A was most likely a theropod.

## Additional Information and Declarations

### Data Availability

The following information was supplied regarding data availability: The footprint measurements (data) are found in Tables 1–4.
